# Morphinism and Narcomania from Opium, Cocain, Ether, Chloral, Chloroform, and Other Narcotic Drugs; Also the Etiology, Treatment, and Medicolegal Relations

**Published:** 1902-07

**Authors:** 


### BOOK REVIEWS.

Morphinism and Narcomania from Opium, Cocain, Ether, Chloral, Chloroform, and other Narcotic Drugs ; also the Etiology, Treatment, and Medicolegal Relations. By T. D. Crothers, M. D., Superintendent of Walnut Lodge Hospital, Conn., Professor of Mental and Nervous Diseases, New York School of Clinical Medicine, etc. 12mo of 351 pages. Philadelphia and London : W. B. Saunders & Co. 1902. [Cloth, $2.00 net.]

After an experience of twenty-five years in the treatment of narcomaniacs, the distinguished author has given us his deductions.

Of greatest interest is the chapter devoted to treatment. He considers first the sudden withdrawal as practised by Levinstein. Of this method he says, it is used in station houses and jails—in private practice it is impracticable. Of the rapid reduction he says: The usual method is to reduce the quantity of morphine taken to two or three grains daily, no matter how much the patient has been in the habit of taking. This method requires special surroundings and trained help. Withdrawal of the drug should be complete in ten days. He advises a gradual withdrawal in those persons in whom there is a strong predisposition to exhaustion from overwork, and have used morphine for relief of the fatigue symptoms.

Of cocainism the author reasserts his claim that the victims are often physicians and professional men. That there is but one way of escape for these drug victims, *i. e.*, to give up everything and make a supreme effort for recovery, then with changed surroundings and a competent physician, the prospect of permanent cure is hopeful.

When he discusses tobacco inebriety, we believe that the personal bias has influenced his statements, and that for instance the following sentence will not receive much support from the professional or lay readers: "The excessive user of tobacco is not an accurate, reliable man in all cases; if he is, it is an exception to the rule." "All men are liars, said St. Paul in his wrath, and that was long before tobacco had impaired man's veracity.

All in all, the book is a helpful one and because of Dr. Crothers's vast experience it will be relied upon as a safe guide in the management of these troublesome cases. J.W.P.

